# Reflecting on Youth Participation in Mental Health Research: Lessons From the *Consell de Joves* in Catalonia

**DOI:** 10.1111/hex.70954

**Published:** 2026-11-03

**Authors:** Matthew Bennett, Ana Lozano‐Sánchez, Claudia Guzmán‐Molina, Constanza Jacques‐Aviñó

**Affiliations:** ^1^ Fundació Institut Universitari per a la Recerca a l'Atenció Primària de Salut Jordi Gol i Gurina (IDIAPJGol) Barcelona Spain; ^2^ Department of Anthropology, Philosophy, and Social Work Universitat Rovira i Virgili Tarragona Spain; ^3^ Network for Research on Chronicity, Primary Care, and Health Promotion (RICAPPS) Barcelona Spain; ^4^ Department of Psychology Universitat Rovira i Virgili Tarragona Spain; ^5^ Equip d'Atenció Primària d'Artesa de Segre, Institut Català de la Salut Artesa de Segre Spain; ^6^ Universitat Autònoma de Barcelona Bellaterra (Cerdanyola del Vallès) Spain

**Keywords:** health equity, mental health, participatory methodology, social justice, youth participatory action research

## Abstract

Participatory approaches in youth and adolescent mental health research are increasingly recognised as important for producing research that is relevant, ethical, and responsive to young people's experiences. However, research remains shaped by adult‐centred structures that can limit young people's influence and reproduce inequities in whose knowledge is recognised. This article presents a reflexive account of the development and implementation of the *Consell de Joves* (CJ), a youth advisory council embedded within a 3‐year mental health research project in Catalonia, Spain (2023–2025). The CJ involved young people across study design, development of research instruments, interpretation of findings, and dissemination. Drawing on established frameworks for youth participation, we reflect on five areas of practice: ethics, safeguarding, and power considerations; recruitment and composition; meeting logistics and participation; youth involvement in research design and dissemination; and ongoing reflection and feedback. We examine how principles of meaningful participation were operationalized, including areas of alignment, tensions, and adaptations. The experience highlighted both the possibilities and limitations of youth participation within institutionally constrained research environments. Flexible engagement, attention to psychological safety, and opportunities for meaningful input supported participation and peer exchange. However, structural barriers related to time, travel, socioeconomic circumstances, and institutional schedules constrained participation. Persistent tensions also emerged around adult–youth power relations, representation, remuneration, and the extent of youth influence within an adult‐led research structure. Reflecting critically on the CJ illustrates how principles of youth participation can be translated into practice while revealing the structural conditions that may constrain their implementation. We provide two practical resources: a reflective journal of recommendations and a step‐by‐step roadmap with critical questions to support more equitable youth participation.

**Patient or Public Contribution:**

Young people participated in the design, conduct, interpretation and dissemination of the broader mental health research project through the *Consell de Joves* (Youth Advisory Council). Council members contributed to shaping research questions, refining interview guides and study materials, interpreting research findings, and advising on dissemination strategies and outputs. They also provided feedback on their experiences of participating in the CJ, which informed the research team's ongoing reflection. However, the present manuscript is a retrospective reflective account developed by the research team. CJ members were not involved in the formal analysis or interpretation of the reflective material concerning the council itself, nor in the development or writing of this manuscript. Their contributions to the broader project and feedback on the CJ experience are acknowledged throughout the paper.

## 1 Background

Participatory methodologies in health research are increasingly recognised as essential for producing findings that are relevant, ethical, and responsive to the populations studied [1, 2, 3]. In the field of youth and adolescent mental health, participatory methodologies have been associated with both improvements in the contextual validity of research findings as well as with benefits for young people themselves, including enhanced agency and wellbeing [4]. As a result, a growing body of literature has examined structured forms of youth and adolescent participation as mechanisms for meaningfully involving young people across research, service, and policy contexts while, in parallel, participatory methods are becoming increasingly institutionalised within many health systems. In the United Kingdom, for example, the National Health Service has a long‐standing tradition of engaging patients and caregivers in research and governance, ensuring that their perspectives shape research priorities, study design, and dissemination [5]. This form of engagement respects ethical rights, strengthens research quality, and enhances transparency in publicly funded research [6].

At the same time, traditional research practices remain deeply adult‐centred. Young people are frequently portrayed as incomplete, lacking agency or moral status, with their experiences filtered exclusively through adults such as parents, guardians, or professionals [7]. This reflects a hierarchy in which colonial epistemologies constructed adulthood as the standard of rationality and objectivity while systematically dismissing young peoples’ knowledge as immature, naïve, or unreliable [8, 9, 10]. Such adultcentrism marginalises young peoples’ voices and yields interventions that miss the complexity of their lives, highlighting the need for research structures that recognise young people as epistemic agents and integrate their perspectives [8, 9]. Therefore, participatory methodologies address an epistemological challenge that emerges when research teams do not include members of the population being studied. In research with young people, this is often masked by the assumption that adults can adequately understand young people's experiences because they were once young themselves. Yet, youth and adolescence are historically and socially situated life stages, and researchers’ own experiences do not reflect the social, cultural, and institutional realities of young people today.

Foundational models such as Hart's Ladder of Participation and Shier's Pathways to Participation have been instrumental in shaping how researchers and practitioners conceptualise the involvement of young people. Hart's Ladder distinguishes between tokenistic and meaningful forms of participation, emphasising that genuine engagement requires young people to move beyond being consulted toward having influence over decisions that affect them [11]. Shier's Pathways builds on this by linking levels of participation to organisational responsibilities, highlighting that youth influence depends on both individual attitudes and institutional structures, policies, and resources that facilitate engagement [12]. Similarly, the Lundy Model of Participation emphasises four interrelated dimensions, space, voice, audience, and influence, underscoring that participation requires opportunities to express opinions, mechanisms for those opinions to be heard, and tangible ways for contributions to shape outcomes [13]. These frameworks provide conceptual tools for understanding and critically reflecting on the quality, ethical integrity, and depth of youth participation, while highlighting the organisational and relational conditions required for meaningful influence.

Recent work has extended these models by foregrounding reflexivity, lived experience, and the everyday dynamics through which participation is enacted. Pavarini et al. [14], for example, offer a reflexive, step‐by‐step model for establishing and sustaining Young People's Advisory Groups, emphasising ethical engagement, representativeness, and ongoing evaluation of impact on both research processes and participants. Similarly, Simmons et al. [15] describe the implementation of an organisation‐wide Youth Engagement and Participation Strategy within a national youth mental health organisation, highlighting youth participation as an evolving, resource‐intensive practice that requires institutional commitment, flexibility, and continuous refinement.

Other frameworks focus explicitly on power, relationships, and inclusion. Jacques‐Aviñó et al. [16] propose a set of reflective questions designed to support inclusive participation across all phases of the research process, encouraging researchers to critically examine whose voices are invited, how decisions are made, and how power is negotiated in practice. In parallel, Frazer et al. [17] conceptualise young people's experiences of participation across three interrelated domains (relationships, power, and personal growth) linked through processes of communication, self‐efficacy, and decision‐making. Importantly, this body of work also demonstrates that participatory methods are never equally accessible. Structural barriers such as time poverty, school and work demands, low self‐efficacy, and inequities in whose knowledge is recognised as legitimate systematically shape who is able to participate [18]. Moreover, power is inevitably present in adult–young person research relationships. Embracing this inevitability with humility and actively reflecting on how it shapes facilitation, engagement, and interpretation is important for fostering ethical, inclusive, and meaningful youth participation [19]. Without such deliberate and sustained efforts, participatory methods with young people risk reproducing existing exclusions even when designed to be inclusive.

Against this backdrop, the *Consell de Joves* (CJ), a youth advisory council, was launched in 2023 as part of a 3‐year, competitively funded project (2023–2025) on youth and adolescent mental health in Catalonia, Spain, supported by Instituto de Salud Carlos III (FIS PI22/01278). Its creation took place in the aftermath of the COVID‐19 pandemic, a period marked by increased mental health consultations in primary care, reduced opportunities for socialisation and collective activity, and fewer spaces for young people to gather, deliberate, and influence decision‐making. In a context where youth mental health had emerged as a pressing public health concern, the CJ was established to create opportunities for young people to contribute to research agendas and processes affecting their lives.

In this reflective practice paper, we examine the development and operation of the CJ, with particular attention to how principles of meaningful youth participation were enacted in practice. We consider tensions and adaptations related to power, accessibility, representation, safeguarding, and youth influence, and draw on these experiences to develop practical considerations and a step‐by‐step roadmap for researchers and organisations seeking to establish meaningful and equitable youth participation.

## 2 Methods

### 2.1 Approach to Reflective Practice

The overarching project within which the CJ was embedded included quantitative and qualitative studies, as well as knowledge‐translation components focused on youth and adolescent mental health. The quantitative studies examined trends, incidence, and factors associated with mental health diagnoses, clinical management, and self‐harm in young people using data from primary healthcare. The qualitative studies explored lived experiences through photovoice and interviews with young people and their families, and focus groups with professionals. The final component focused on co‐creating audiovisual dissemination materials for the public and key stakeholders. The CJ acted as a cross‐cutting participatory mechanism, providing structured opportunities for young people to contribute to study design, data collection, interpretation, and dissemination across the different components of the project.

As the research team responsible for designing, coordinating, and facilitating the CJ, we adopted a reflective practice approach to critically examine our experiences and decisions within their situated context, including how our assumptions, relationships, and institutional conditions shaped participation [20, 21]. We reflect on how principles of meaningful youth participation were translated into practice, including where they aligned with established frameworks and guidance, where tensions emerged, and how the research team adapted its approach in response. Reflection was informed by established approaches to youth participation, including Hart's Ladder of Participation [11], Shier's Pathways to Participation [12], the Lundy Model of Participation [13], and more recent guidance emphasising reflexivity, power, inclusion, safeguarding, and co‐production [14, 16, 22, 23]. These frameworks were used as conceptual reference points for examining how participation was enacted, where practice aligned with or diverged from established principles, and what tensions and adaptations emerged.

The reflective process drew on three complementary sources: (1) participant feedback collected during and at the end of CJ activities; (2) observations and experiences of the research team during facilitation and interaction with CJ members; and (3) reflexive discussions among research team members. Reflection occurred at two interconnected stages. Throughout the implementation of the CJ, the research team discussed emerging observations, participant feedback, and challenges to inform ongoing decisions and adaptations. Following the conclusion of the initiative, the team revisited these experiences alongside the collected feedback, including the anonymous end‐of‐initiative survey, to identify recurring tensions, adaptations, and lessons. These reflections were considered in relation to established youth participation frameworks and synthesised into the practical considerations presented in Table 1 and the roadmap in Figure 1.

The present paper was developed retrospectively by the research team and was not co‐designed or co‐authored with CJ members. This decision also reflects a broader structural and ethical tension in that participatory processes such as youth advisory councils are not consistently recognised within research funding schemes, making it difficult to commit to sustained youth participation before funding is secured. Once funding was obtained, the CJ was established as part of the broader research project. The manuscript therefore represents the research team's critical reflection on its development and operation, drawing on feedback provided by CJ members during the initiative. CJ members were not involved in the formal analysis or interpretation of the reflective material presented here.

### 2.2 Team Reflexivity and Positionality

The reflective process was also informed by the disciplinary and epistemological positioning of the research team. The team comprised professionals from social work, anthropology, psychology, and social paediatrics, with experience in qualitative and participatory research and critical approaches to health and social inequities. As a team of adults who had experienced adolescence but were no longer part of the youth population at the time of the initiative, we also recognised the generational distance between researchers and CJ members. These perspectives informed how the team conceptualised youth participation, power, knowledge, and the role of experiential expertise within research. In particular, our work is informed by critical perspectives that question the dominance of biomedical and positivist approaches to health research and recognise knowledge as situated within social, cultural, and institutional contexts. This positioning shaped both the design and facilitation of the CJ and the subsequent interpretation of experiences and challenges. We therefore approached reflection not as a neutral assessment of the initiative, but as a situated process shaped by our disciplinary perspectives, assumptions, and relationships with CJ members [24].

## 3 Key Dimensions of Youth Participation in Practice

The following sections examine five interconnected dimensions of establishing and operating the CJ: (1) Ethics, safeguarding, and power considerations; (2) Recruitment and composition; (3) Meeting logistics and participation; (4) Shaping research design and dissemination through CJ input; and (5) Ongoing reflection and feedback. Although presented separately for clarity, these dimensions were interconnected throughout the initiative rather than representing sequential stages.

### 3.1 Ethics, Safeguarding and Power Considerations

Given the involvement of young people and the focus on mental health, safeguarding and ethical considerations were important methodological components of this initiative. Established guidance on youth participation emphasises that meaningful participation requires more than formal consent. Meaningful participation also requires attention to psychological safety, voluntary engagement, power relations, and the conditions under which young people can exercise influence [13, 22]. We therefore approached ethics and safeguarding as ongoing dimensions of participation in addition to a fundamental requirement addressed during recruitment.

The establishment and operation of the CJ underwent institutional ethical review by the IDIAP Jordi Gol Ethics Committee (Barcelona, January 17, 2023; code 22/206‐P). Although CJ members were involved as contributors to the research process, ethical oversight was considered important to ensure appropriate safeguards, informed participation, and procedures for managing potential risks associated with participation. Written informed consent was secured from all participants, including consent for audiovisual recording. For participants under 16 years of age, parental or guardian consent was obtained alongside members’ assent, in accordance with local legislation governing research with young people.

Beyond formal ethical requirements, the initiative was guided by safeguarding frameworks for youth and adolescent participation and co‐design [22], which emphasise psychological safety, clear communication, flexibility, and ongoing attention to power dynamics. Participation was framed as voluntary, negotiable, and revisable over time. Prior to joining the council, members were clearly informed about its purpose, expected modes of involvement, and the nature of discussions. CJ members could modify their level of engagement or withdraw at any point without consequence. Particular care was taken to avoid implicit or explicit pressure to disclose personal or lived experiences related to mental health. Contributions were invited at a level determined by each participant, and the sharing of sensitive information was neither expected nor encouraged.

Power relations and remuneration constituted an additional axis of ongoing reflexivity. As highlighted by Guo et al. [22], financial compensation (or its absence) shapes who is able to participate and how participation is experienced. Due to funding constraints, formal remuneration was not provided. To partially mitigate the resulting imbalances, the research team sought to minimise demands on participants’ time, offered flexible modes of engagement, ensured transparent acknowledgment of contributions in dissemination outputs, and created opportunities for skills development, mentorship, and exposure to research processes. Nonetheless, we recognise that the absence of financial compensation likely constrained participation for some members, particularly those experiencing socioeconomic precarity, and may have inadvertently reproduced existing participation inequities.

### 3.2 Recruitment and Composition

Established participation frameworks emphasise that meaningful involvement depends not only on inviting young people to participate but also on considering who is able to participate and whose perspectives may remain excluded [12, 18]. In the case of the CJ, the only formal inclusion criterion was age (12–24 years). Recruitment emphasised participants’ motivation and willingness to remain engaged over 3 years, as sustained participation was voluntary and unpaid and therefore depended largely on genuine interest in the topic and willingness to contribute to collective reflection. Members were identified through snowball and convenience sampling, via personal recommendations from research team members with prior clinical or community contact. Open calls circulated via social media and university campuses were less effective, while personal recommendations proved far more successful, as pre‐existing trust facilitated both recruitment and sustained participation.

Scheduling and communication were primarily managed through phone calls and WhatsApp messages. Each potential participant was contacted individually to explain the project, address any questions, confirm interest prior to the first meeting, and provide more details to facilitate travel and logistics. Additionally, for participants under 18, the project team first contacted their parents or guardians to explain the study and obtain parental consent, in addition to securing the assent of the participant themselves. Recruitment procedures were designed to ensure informed, pressure‐free participation, with particular attention to consent, assent, and safeguarding for participants under 18.

A total of 20 young people participated in the CJ over the 3‐year period. Of these, 14 were recruited at the outset, while 6 joined subsequently. Participation varied over time, with 18 participants remaining actively engaged at the final stage. Changes in participation were primarily related to school, university, work, mobility, and other personal circumstances. The CJ was divided into two age groups (12–16 and 17–24 years) to reflect distinct developmental stages, facilitate shared experiences, minimise hierarchical dynamics, and ensure age‐appropriate discussions.

Participants varied in gender and, to a lesser extent, race and family migratory background. Recruitment was most successful among young people with academic capital or proximity to the university environment, such as psychology students, reflecting how access to time, resources, and institutional networks shapes participation. As a result, some socioeconomic groups were underrepresented, and retention of participants from more resource‐constrained backgrounds was limited. While broad socioeconomic inclusion was not a primary objective of this initiative, these observations highlight that if equitable representation across contexts is a priority, recruitment strategies and participation formats may need to be adapted. Possible strategies may include outreach through community organisations, flexible scheduling, and additional support to reduce barriers for young people with limited time or resources.

### 3.3 Meeting Logistics and Participation

Participation frameworks emphasise the importance of creating spaces in which young people have genuine opportunities to express themselves and influence discussions [13]. In practice, however, the organisation of participatory spaces remained partly determined by institutional constraints. CJ meetings were held approximately twice per year, each lasting 2 h and scheduled outside academic hours. The meetings took place in the research centre's meeting room in the centre of Barcelona. This setting was chosen with care because participation spaces are not neutral and reflect power dynamics that influence who feels welcome and able to attend [25]. The central location and free venue were intended to facilitate access, although their accessibility varied according to participants’ place of residence, available resources, and mobility.

All research team members contributed to planning and facilitation, emphasising discussion‐based activities, co‐creation exercises, and collaborative reflection on study instruments and preliminary results. Meetings were facilitated with attention to psychological safety, clear boundaries around sharing personal experiences. In the first meeting, the group held a dedicated discussion to collectively agree on how work would be conducted and to establish guidelines for confidentiality, co‐developing ground rules that defined participation dynamics, needs, and expectations. These ground rules emphasised respect and confidentiality, while participants were informed that confidentiality could not be guaranteed in circumstances involving safeguarding concerns or serious risk of harm, in accordance with institutional safeguarding procedures. Input was then gathered through ongoing group discussions, written feedback, and co‐creation of materials.

Participation in the CJ was entirely voluntary, with no minimum attendance required. Individual contact with council members allowed them to participate according to their circumstances, temporarily pausing involvement when necessary without penalty. To make participation feasible for everyone, the research team maintained constant, open communication via an official WhatsApp account created exclusively for the project, allowing members to share thoughts, reflections, and feedback at any time. In addition to in‐person meetings, participants could engage through online activities, such as Google Forms, and meeting resources and materials were shared so feedback could be submitted even if they could not attend. This flexible approach resisted institutional exclusion and emphasised that participation should be accessible and adaptable to individual needs.

Despite efforts to promote flexibility and accessibility, meeting schedules, locations, and session dynamics were largely established by the adult research team. CJ members were offered multiple options for days and times and could vote on their preferred slots, but these options were all shaped by the researchers’ availability rather than emerging from the participants’ own schedules. While this approach facilitated coordination within institutional constraints, it also reflects adult‐led, predefined participation parameters.

Participatory mechanisms like the CJ can involve discussions of sensitive topics, including mental health, discrimination, precarity, or violence. During the CJ meetings, the research team therefore remained attentive to signs of emotional discomfort and to the boundaries participants set around sharing personal experiences. All sessions were facilitated by professionals from psychology, social work, and medicine, and informal debriefing and individual follow‐up were available when needed. No participant requested formal individual follow‐up. These arrangements were considered important by the research team to create conditions in which participants could engage with sensitive topics without being expected to disclose personal experiences.

The research team also observed that group composition appeared to shape participation during discussions of sensitive topics. For example, in a session in which findings from the broader research project identified racism as a factor associated with distress, the discussion was led primarily by non‐racialized participants. Although we cannot determine whether this pattern was directly related to participants’ social positions, as no formal assessment was conducted, it prompted reflection within the research team about how group composition and perceived power differences may influence willingness to speak when topics directly relate to participants’ lived experiences. This experience also led us to recognise that not all topics need to be addressed collectively. When issues may particularly implicate participants’ own experiences or identities, individual or smaller, safer spaces may provide more appropriate opportunities for reflection and contribution. This informed our subsequent emphasis on facilitation strategies that attend to power and representation and allow for different ways of participating.

### 3.4 Shaping Research Design and Dissemination Through CJ Input

The CJ contributed to study design, instruments, interpretation of results, and dissemination. Through these activities, CJ members raised issues and perspectives that the research team considered when refining study materials, interpreting findings, and developing dissemination outputs. Recognised as the true experts of their own lives, contexts, and environments [26], CJ members contributed experiential perspectives that complemented the clinical, methodological, and academic expertise of the research team. We regarded these contributions as an important form of expertise in shaping the research process, while recognising that CJ members and researchers occupied different institutional positions, roles, and levels of decision‐making power. Their contributions informed specific research decisions, including the development of qualitative instruments, interpretation of findings, and dissemination materials.

For qualitative studies, the CJ co‐designed and refined interview scripts, optimising language, question order, prompts, and response formats to ensure clarity and relevance. To elicit their input, members worked in small groups to generate questions they would want to ask a peer of the same age with a mental health diagnosis, discussing and collectively writing the most meaningful and accessible items. Their input was particularly valuable given that the interviews were conducted with peers, allowing the scripts to be tailored in a way that enhanced accessibility, comprehension, and authenticity of responses among adolescent participants.

For quantitative studies, CJ members provided critical interpretations of results, highlighting peer dynamics, online interactions, and nuanced family expectations. To facilitate this, results were presented conceptually and schematically so that CJ members could engage in more detail with the key findings. They were then asked to share which patterns stood out to them, whether the findings reflected their lived experiences, and how they would prefer the results to be presented. Their feedback directly informed the analysis, reporting, and organisation of results and discussion sections in publications.

CJ members also informed project dissemination, advising on video creation, language, visuals, and communication strategies to resonate with young people. They participated in a recording session, offering an authentic view of CJ dynamics for inclusion in dissemination materials. Through these contributions, the CJ helped ensure that both the research process and outputs reflected youth priorities and perspectives, illustrating how youth input was incorporated into different stages of the research process.

While CJ input was carefully taken into account and incorporated throughout the entire research process, feedback was most often solicited at discrete moments (e.g., on specific products or outputs), rather than through a structured, iterative feedback loop sustained across the full research process. As a result, opportunities to explicitly revisit how CJ contributions were taken up, adapted, or constrained in final decisions were limited and may have reduced the sense of continuous participation. Future implementations would benefit from explicit feedback loops that document, revisit, and communicate how youth input informs evolving research decisions.

The CJ was acknowledged in dissemination materials and in all published scientific articles to which they contributed feedback and expertise in interpreting study results. Moreover, members who were interested in pursuing their own research on mental health, such as for school assignments, were also provided with guidance and contacts to support these projects, fostering their agency and skills as emerging researchers.

### 3.5 Ongoing Reflection and Feedback

At the end of each in‐person session, facilitators conducted a brief verbal check‐in with participants before the group dispersed. Participants were invited to share, if they wished, how they were feeling following the session and whether there was anything they wanted the research team to know or follow up on. These check‐ins were conducted informally rather than as a structured assessment and did not involve systematic collection of psychological or clinical data. The approach was informed by principles of Gestalt practice, particularly attention to present‐moment experience, awareness, and reflection on participants’ immediate responses to the interaction [27]. Individual follow‐up could subsequently be arranged through direct communication with a member of the research team when needed, though no participant requested formal individual follow‐up during the initiative.

At the conclusion of the initiative, CJ members were invited to complete an anonymous online feedback survey developed by the research team for this initiative. The survey included four closed‐ended questions and eight open‐ended questions addressing participants’ overall experience, perceived influence, comfort, valued aspects of participation, activities, barriers, perceived benefits, and suggestions for improvement (Supplementary Material S1). The survey was not a validated evaluation instrument and was used to inform reflexive discussion rather than as a standalone process evaluation. No identifying or sociodemographic information was collected, and the anonymous design therefore did not permit characterisation of respondents.

Participants generally described their experience positively. The mean rating for overall experience was 4.8 on a 1–5 scale, and all respondents indicated that their opinions and ideas were listened to and taken into account. Reflections highlighted the value of exchanging perspectives with peers and adults, having a supportive space to discuss difficult or stigmatised topics, and receiving clear organisation and facilitation. Participants also identified areas for improvement, including more frequent meetings, more regular updates on research progress, and difficulties participating alongside school, extracurricular, and work commitments.

These reflections reinforced an important tension within the initiative. Flexibility and voluntary participation were valued, but structural constraints continued to shape who could participate consistently. In response, the team supplemented in‐person meetings with online activities, maintained individualised communication via WhatsApp, and allowed participants to pause or resume involvement according to their circumstances. These adaptations improved flexibility but did not eliminate the structural barriers affecting participation.

The reflective process also required consideration of the researchers’ own positionality. The research team consisted of adult professionals with academic and clinical backgrounds who simultaneously designed, facilitated, and interpreted the initiative. Their age, institutional positioning, and perceived expertise inevitably shaped participation dynamics and the interpretation of feedback. Anonymous feedback reduced, but could not eliminate, the possibility of social desirability bias. Regular reflexive discussions within the team therefore considered how assumptions, expectations, and investments in the initiative might influence interpretation, while ensuring that participant‐identified challenges were considered alongside positive assessments.

## 4 Discussion

### 4.1 Team Reflections, Challenges and Lessons Learned

Participatory methodology with young people requires sustained attention to the adult‐centred structures that can constrain meaningful involvement. While numerous frameworks for youth participation and co‐production have been proposed, there remains limited consensus on how these approaches are enacted in practice, particularly within mental health research [7, 28]. Recent reviews highlight that participatory methods frequently adopt co‐design principles unevenly, shaped by practical constraints such as time, recruitment, and institutional expectations [29]. Reflecting on the CJ experience illustrates the complexity of translating principles of meaningful participation into an institutional research setting. The initiative created opportunities for young people to contribute perspectives on their lives, social worlds, and priorities, while simultaneously revealing the structural and relational conditions that shaped the extent to which those contributions could influence research processes.

Both current evidence and our reflections on the CJ suggest that power dynamics and accessibility shaped how participation was experienced and sustained [6, 18]. These reflections were informed by observations of participation during meetings, patterns of engagement over time, and feedback provided by CJ members. This distinction is important because the initiative was not designed to measure the effects of particular participatory strategies. Rather, examining these experiences retrospectively allowed the research team to identify tensions between the principles of equitable participation and the institutional conditions within which the CJ operated.

Consistent with recent syntheses of youth engagement in mental health research, young people with greater self‐efficacy, flexible schedules, or access to material and social resources were more able to participate consistently, while others faced structural barriers related to socioeconomic circumstances, mobility, and competing responsibilities [17, 29, 30]. For example, some members from rural areas were unable to attend in‐person meetings, illustrating how logistical constraints can contribute to the overrepresentation of urban‐based participants despite inclusive recruitment intentions. Our attempts to increase flexibility through different participation formats and individualised communication reduced some barriers but could not overcome the wider structural conditions shaping participation. This experience reinforced that accessibility is also a question of whose circumstances research structures are designed to accommodate.

Retention and continuity also prompted reflection on the participation of young people occupying minority or otherwise marginalised positions within the group. The research team observed that some racialized participants and participants from lower socioeconomic backgrounds participated less consistently or paused their involvement over time. These patterns coincided with work, school, mobility, and other personal circumstances, and changes in participation cannot be attributed to any single factor. Nevertheless, they prompted reflection on what happens when participants’ experiences or circumstances differ from those of the majority of the group. Similar concerns have been described in youth participatory action research, where power relations and structural inequalities can influence whose voices are heard and acted upon [31, 32].

From a feminist standpoint, social positions shape what people are able to observe and understand, meaning that perspectives rooted in marginalisation can illuminate structural dynamics that dominant positions may overlook [33]. Yet, the presence of marginalised voices does not guarantee that those perspectives will be expressed or heard. Our experience therefore suggests that equitable participation requires intentional facilitation and support structures that attend to representation, power, and institutional trust, particularly given that some communities may have reasons for mistrust toward scientific and research institutions [34]. In practice, this may include creating multiple ways to contribute, checking whose perspectives are being heard during discussions, and considering whether some topics or forms of participation would be better supported through smaller‐group or individual engagement.

From an ethical and methodological perspective, the CJ also highlighted the importance of critically examining researchers’ roles as facilitators within participatory processes. Lloyd et al. [23] emphasise “scene setting,” cyclical engagement, and appropriate recognition of young people expertise as components of meaningful co‐production. In practice, the research team attempted to operationalise these principles by providing age‐appropriate opportunities to develop research literacy, creating structured but flexible spaces for dialogue, and involving young people in dissemination activities. At the same time, the process revealed a persistent tension between youth influence and institutional control. Although CJ members could shape instruments, interpretations, and dissemination materials, researchers retained responsibility for study timelines, methodological decisions, and final outputs. Similarly, CJ feedback was often solicited at discrete points rather than through continuous feedback loops, limiting opportunities to revisit how suggestions were incorporated or why particular decisions were not adopted. These experiences suggest that meaningful influence requires not only opportunities to provide input, but also transparent mechanisms for communicating how that input affects subsequent decisions.

Moreover, an important ethical tension concerns the positioning of young people involved in advisory councils. Institutional ethical review can implicitly frame young advisors primarily as research participants, even when their intended role is that of contributors or co‐producers of the research process. In the CJ, we considered ethical oversight necessary because participants included minors and because discussions could involve sensitive topics related to mental health. However, ethical approval did not by itself resolve the question of how young people should be positioned within the research team or how their experiential expertise should be recognised. Future initiatives should therefore distinguish between the protective function of ethical oversight and the participatory status of youth collaborators, avoiding approaches in which ethical procedures inadvertently reinforce adult–youth hierarchies or reduce youth involvement to that of research subjects.

The experience also raised questions about recognition, remuneration, and the risk of academic extractivism. Reviews of co‐production practices identify remuneration, diversity, and safeguarding as persistent challenges, particularly when participation is voluntary and unfunded [23]. In the CJ, the absence of financial compensation was a consequence of funding constraints, and although we attempted to mitigate this through flexible participation, acknowledgment of contributions, skills development, mentorship, and opportunities to engage with research processes, these measures cannot be considered equivalent to remuneration. The experience therefore reinforced that recognition and compensation are distinct and that voluntary participation may systematically favour young people with greater material and temporal resources.

Sustainability raises a related question surrounding what happens to a youth advisory council when the research project that created it ends. Designing structures that allow youth groups to continue autonomously, if desired, represents one possible way to counter extractive dynamics in which young people's time and knowledge primarily serve academic purposes [35, 36]. Supporting autonomous continuation requires researchers to relinquish some control and remain responsive to youth priorities beyond the immediate needs of a funded project. For future initiatives, this suggests that sustainability and autonomy should be considered from the outset rather than treated as an end‐of‐project consideration.

Finally, the physical and institutional setting of participation warrants attention. Although the CJ meetings were held in a centrally located research centre with the intention of facilitating access, the setting remained an academic environment shaped by adult researchers and institutional norms. Participation spaces are not neutral and can influence who feels welcome, comfortable, and entitled to speak [25]. This experience suggests that future initiatives could consider community‐based or non‐institutional spaces, where feasible, alongside virtual and asynchronous formats. More broadly, leveraging institutional resources while remaining attentive to how these resources shape participation may help create spaces in which young people can exercise greater ownership of the process.

Based on the three sources of reflection described above, including participant feedback, research‐team observations and experiences, and reflexive discussions among team members, Table 1 synthesises the main principles, practices, tensions, and lessons that emerged across the five areas of the CJ's operation. These were considered alongside the youth participation frameworks and guidance that informed the initiative [11, 12, 13, 14, 16, 22, 23]. The “Reflections and implications” column translates these observations into practical considerations for researchers planning or sustaining a youth advisory council. The “Reflections and implications” column translates these observations into practical considerations for researchers planning or sustaining a youth advisory council. Complementing this, Figure 1 translates these lessons into a practical roadmap, highlighting key steps and critical questions at each stage of the process. Together, these outputs function both as a record of our initiative and as structured tools to guide others seeking to engage young adults meaningfully in research.

**Table 1:** Reflective lessons and practical considerations for establishing and sustaining a Youth Advisory Council.

Dimension	Participation principle	What we did in practice	Tensions/challenges encountered	Reflections and implications
(1) Ethics, safeguarding & power	Voluntary participation; psychological safety; attention to power	Clear roles; consent/assent; co‐created ground rules; no pressure to disclose	Adult–youth power; no remuneration; risk of extractive participation	Safeguarding must extend beyond formal ethics; examine who may be excluded by unpaid participation
(2) Recruitment & composition	Inclusion and representation	Snowball/open recruitment; age groups; individual contact	Limited socioeconomic diversity; retention barriers	Inclusion at recruitment does not guarantee equitable participation
(3) Logistics & participation	Accessible space; opportunities for voice	Flexible scheduling; online/asynchronous options; WhatsApp	Adult‐led scheduling; travel; uneven participation	Flexibility helps but cannot eliminate structural barriers
(4) Research design & dissemination	Voice, audience, influence	Co‐designed instruments; interpretation; dissemination	Influence bounded by institutional decisions; discrete feedback	Youth input requires explicit feedback loops and transparency about decisions
(5) Reflection & feedback	Reflexivity and accountability	Session check‐ins; anonymous feedback; team reflection	Social desirability; researchers’ dual role	Reflection should be continuous and include researchers’ positionality
Sustainability	Recognition, autonomy, non‐extractivism	Acknowledgment; mentorship; consideration of continuation	No remuneration; project‐bound structure	Plan sustainability and autonomy from the outset

*Source:* Author's own creation

**Figure 1:**
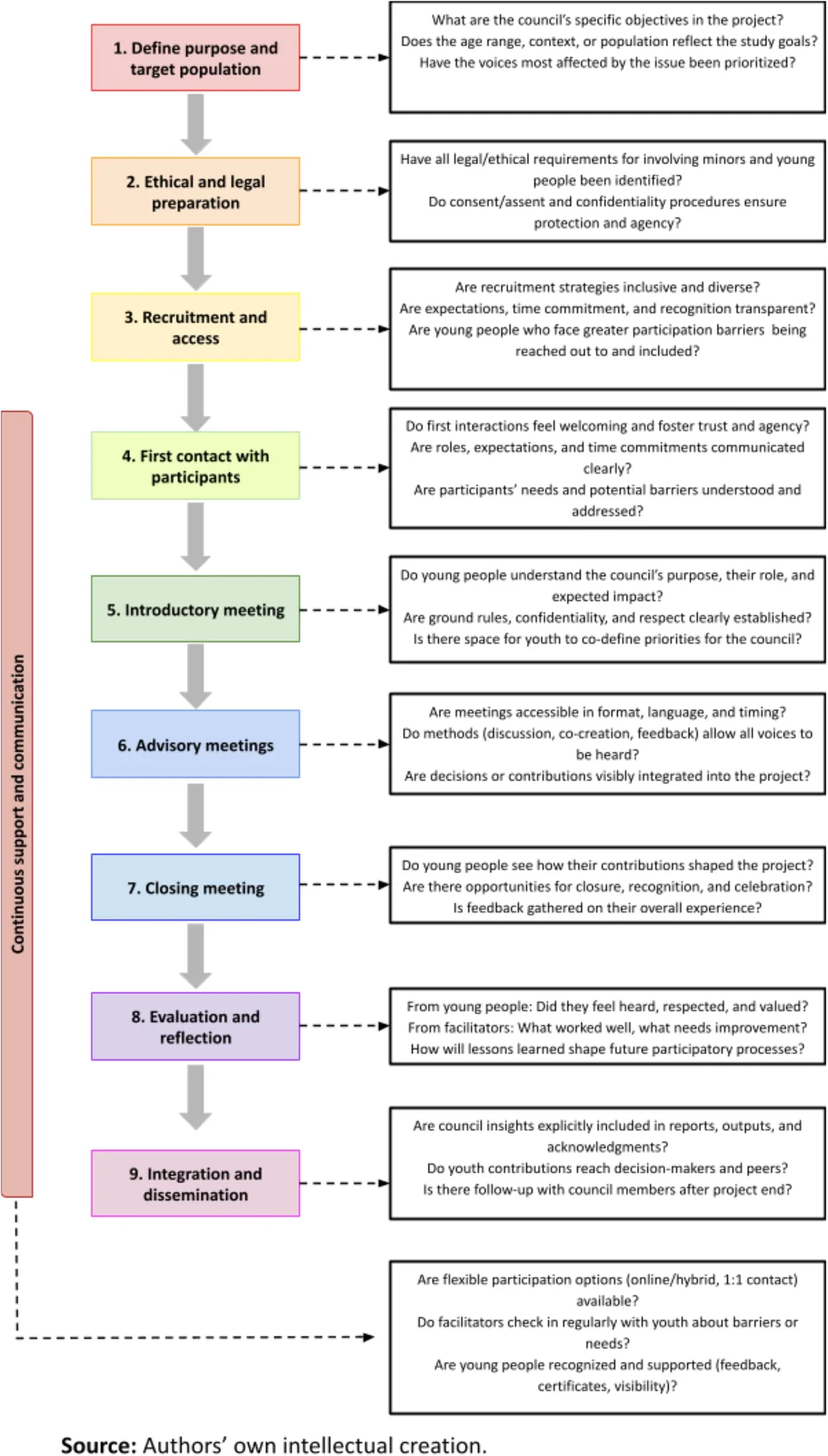
Roadmap for the creation and execution of youth advisory councils.

## 5 Strengths and Limitations

A strength of this account is its longitudinal perspective across the development and operation of the CJ, allowing reflection on participation as an evolving process rather than as a single intervention or endpoint. The use of multiple sources of reflection, including facilitator observations and discussions, informal participant feedback, anonymous survey responses, and written reflections from CJ members, provided complementary perspectives that informed the research team's interpretation of the initiative. The integration of youth perspectives into the interpretation of research findings and dissemination further strengthened the participatory nature of the initiative.

Several limitations should nevertheless be considered. First, the research team was directly involved in designing, facilitating, observing, and reflecting on the CJ, and therefore the account cannot be considered independent of the researchers’ perspectives and positionality. Although multiple sources of feedback were considered, including extensive qualitative feedback collected throughout the CJ, anonymous end‐of‐initiative feedback, and ongoing team reflection, these sources remain situated within the relationships and institutional context in which the CJ operated. Second, because the end‐of‐initiative survey was intentionally anonymous, we were unable to characterise respondents or examine whether feedback differed across sociodemographic groups. This was considered appropriate to facilitate candid feedback, but limits our ability to assess whose experiences were represented in the survey responses. Finally, the CJ did not fully reflect the diversity of young people in Catalonia, particularly in relation to socioeconomic background, geographic location, and racialization. From an equity perspective, this also highlights the need to consider how recruitment and engagement strategies can reach young people who may be less likely to participate because of stigma, institutional mistrust, or previous experiences with research and other institutions. These limitations reinforce that the account should be understood as a situated reflection on one youth advisory council rather than a comprehensive or generalisable assessment of youth participation.

## 6 Conclusion

Meaningful youth participation requires ongoing attention to power, accessibility, equity, safeguarding, and the conditions under which young people can influence research. Reflecting on the development and operation of the CJ illustrates both the possibilities and the limits of translating established principles of youth participation into an institutionally structured research project. The experience highlighted that flexibility, psychological safety, and meaningful opportunities for contribution can support participation, while also revealing persistent tensions around adult control, inequitable access to participation, remuneration, and continuity.

Our experience with the CJ in Catalonia offers a set of practical and critical considerations for researchers seeking to develop youth advisory councils. The reflective journal and roadmap provided in this paper are intended to support teams in asking not only whether young people have been invited to participate, but whose participation is enabled, whose voices are heard, how influence is exercised, and how power is negotiated throughout the research process. Importantly, young people involved in advisory councils should not be understood as representative of all young people; their contributions instead provide situated forms of experiential knowledge that can challenge assumptions and contextualise research findings. Meaningful participation therefore requires continuous reflexivity, including awareness of researchers’ own positioning as adults within adult‐led research institutions, alongside institutional commitment and willingness to adapt established research practices in response to young people's perspectives.

## Author Contributions

**Matthew Bennett:** conceptualisation, visualisation, investigation, methodology, writing – original draft, writing – review and editing. **Ana Lozano‐Sánchez:** investigation, writing – review and editing, methodology, visualisation. **Claudia Guzmán‐Molina:** investigation, methodology, writing – review and editing. **Constanza Jacques‐Aviñó:** conceptualisation, funding acquisition, investigation, methodology, writing – review and editing, visualisation.

## Ethics Statement

The initiative adhered to the Declaration of Helsinki and Good Research Practice guidelines, with data processed under the EU GDPR (2016/679). Approval was granted by the IDIAP Jordi Gol Ethics Committee (Barcelona, January 17, 2023; code 22/206‐P). Written informed consent was obtained from all participants, including audio recording permission; for participants under 18, both parental consent and youth assent were required.

## Conflicts of Interest

The authors declare no conflicts of interest.

## Supporting information


Supporting File

## Data Availability

This reflective practice paper shares the process and outputs of a youth advisory council initiative. It does not involve primary data collection or produce shareable datasets. Hence, no data are available.
