# Supplementary material for: Reflecting on Youth Participation in Mental Health Research: Lessons From the Consell de Joves in Catalonia
Source: Health Expect. 2026 Nov 3;29(6):e70954. doi: 10.1111/hex.70954 (PMC13729264; doi:10.1111/hex.70954)
Supplement: Supplementary file 1 [file HEX-29-e70954-s001.pdf]

Supplementary Material S1. Anonymous feedback survey used with *Consell de Joves* members.

The following anonymous survey was administered online at the conclusion of the initiative. The survey was developed by the research team for the purpose of obtaining participant feedback and informing reflexive discussion about the operation of the Consell de Joves. It was not intended as a validated evaluation instrument. No sociodemographic or identifying information was collected. Closed-ended responses were summarized descriptively, while open-ended responses were reviewed by the research team to identify recurring issues and inform reflection on the initiative.

#	Question (original Catalan)	English translation	Response format
1	En una escala de l'1 al 5, com valoraries la teva experiència global al Consell de Joves?	On a scale from 1 to 5, how would you rate your overall experience in the Youth Advisory Council?	1–5 scale
2	Has sentit que les teves opinions i idees són escoltades i tingudes en compte?	Have you felt that your opinions and ideas were listened to and taken into account?	1–5 scale
3	T'has sentit còmode/a participant a les reunions i activitats?	Have you felt comfortable participating in the meetings and activities?	1–5 scale
4	Què és el que més t'ha agradat de formar part del Consell de Joves?	What did you like most about being part of the Youth Advisory Council?	Open-ended
5	Quins aspectes de les reunions, activitats o comunicació creus que han funcionat millor?	Which aspects of the meetings, activities, or communication do you think worked best?	Open-ended
6	Quina activitat t'ha agradat més? Per què?	Which activity did you like most? Why?	Open-ended
7	Què es podria millorar perquè el Consell de Joves sigui una experiència millor per a tu i per a altres persones?	What could be improved to make the Youth Advisory Council a better experience for you and others?	Open-ended
8	Quina activitat t'ha agradat menys? Per què?	Which activity did you like least? Why?	Open-ended
9	Has tingut algun obstacle (temps, transport, escola/feina, etc.) que hagi fet difícil participar?	Have you experienced any barriers (e.g., time, transport, school/work) that made it difficult to participate?	Open-ended

10	Recomanaries participar en un consell de joves com aquest a altres persones?	Would you recommend participating in a youth advisory council like this to other people?	Yes/No
11	Què és el que t’ha aportat participar al Consell de Joves?	What has participating in the Youth Advisory Council brought to you?	Open-ended
12	Tens algun altre comentari o suggeriment que vulguis compartir?	Do you have any other comments or suggestions you would like to share?	Open-ended
